# Crystal structures of H-2D^b^ in complex with the LCMV-derived peptides GP92 and GP392 explain pleiotropic effects of glycosylation on antigen presentation and immunogenicity

**DOI:** 10.1371/journal.pone.0189584

**Published:** 2017-12-18

**Authors:** Ida Hafstrand, Daniel Badia-Martinez, Benjamin John Josey, Melissa Norström, Jérémie Buratto, Sara Pellegrino, Adil Doganay Duru, Tatyana Sandalova, Adnane Achour

**Affiliations:** 1 Science for Life Laboratory, Department of Medicine Solna, Karolinska Institutet, and Department of Infectious Diseases, Karolinska University Hospital, Solna, Stockholm, Sweden; 2 NSU Cell Therapy Institute, Nova Southeastern University, Fort Lauderdale, FL, United State of America; 3 College of Allopathic Medicine, Nova Southeastern University, Fort Lauderdale, FL, United State of America; 4 DISFARM, Dipartimento di Scienze Farmaceutiche, Sezinone Chimica Generale e Organica, Università degli Studi, Milano, Italy; University College London, UNITED KINGDOM

## Abstract

Post-translational modifications significantly broaden the epitope repertoire for major histocompatibility class I complexes (MHC-I) and may allow viruses to escape immune recognition. Lymphocytic choriomeningitis virus (LCMV) infection of H-2^b^ mice generates CD8^+^ CTL responses directed towards several MHC-I-restricted epitopes including the peptides GP92 (CSANNSHHYI) and GP392 (WLVTNGSYL), both with a N-glycosylation site. Interestingly, glycosylation has different effects on the immunogenicity and association capacity of these two epitopes to H-2D^b^. To assess the structural bases underlying these functional results, we determined the crystal structures of H-2D^b^ in complex with GP92 (CSANNSHHYI) and GP392 (WLVTNGSYL) to 2.4 and 2.5 Å resolution, respectively. The structures reveal that while glycosylation of GP392 most probably impairs binding, the glycosylation of the asparagine residue in GP92, which protrudes towards the solvent, possibly allows for immune escape and/or forms a neo-epitope that may select for a different set of CD8 T cells. Altogether, the presented results provide a structural platform underlying the effects of post-translational modifications on epitope binding and/or immunogenicity, resulting in viral immune escape.

## Introduction

The impact of post-translational modifications (PTMs) on disease progression is now well established [1–3]. PTMs such as deamidation [4], cysteinylation [5], nitrotyrosination [6,7], glycosylation [8,9] or phosphorylation [10,11] alter the epitope repertoire in a large ensemble of diseases including cancers [12–14], autoimmune disorders [15,16] and viral infections [17,18]. PTMs may result in the generation of disease-specific immunogenic neo-epitopes [19] and/or allow for immune escape [20]. Glycosylation is one of the most common PTMs, with important consequences for MHC-I and MHC-II presentation, and consequently T cell recognition [21–24]. There are commonly three types of constitutive glycans [19,22,25]. While N-linked glycans use a N-acetylglucosamine (GlcNAc) sugar moiety that binds to the side chain of the asparagine residue within the conserved motif Asn-X-Ser/Thr where X ≠ Pro, O-linked glycans bind to serine or threonine residues via a sugar moiety such as GlcNAc without any consensus sequence pre-requirement. The third type of glycosylation includes the carbohydrate components of glycosylphosphatidylinositol (GPI) anchors. T cell responses against O-glycosylated proteins can be significantly influenced by the nature and the position of peptide-attached sugar moieties [9,19,22,26]. N-linked glycosylation can also give rise to secondary de-glycosylation, resulting from the conversion of asparagine to aspartate [27], which ultimately means that a potentially N-linked glycosylation site can exist in three different forms, wild-type (WT), glycosylated (GlcNAc) and deglycosylated (D).

The addition of *N*-linked glycans to nascent polypeptides occurs co-translationally in the endoplasmic reticulum (ER), as part of their maturation [28]. The glycosylation state serves as an indicator for a large amount of proteins, allowing the ER quality control system to monitor the conformation and the appropriate fold of these polypeptides. Proteins that are inadequately folded are often dislocated to the cytoplasm, where they are subjected to proteasomal degradation. Specific recognition and elimination of tumors and/or pathogen-infected cells by cytolytic CD8^+^ T lymphocytes (CTLs) critically relies on the presentation of antigenic peptides by MHC class I molecules (MHC-I) at the cell surface. The antigenic peptide repertoire is generated from mature and immature proteins (defective ribosomal products, DRiPs), which depends mainly on degradation by the proteasome during or after their synthesis [29,30]. Since many antigens are glycoproteins, including tumor-associated and pathogen-derived molecules, the glycosylation status of these epitopes may affect their processing and the amount that is presented on the cell surface [31].

Although the *de-facto* MHC-I presentation of N-glycosylated peptides on the surface of healthy, stressed or infected cells has still not be proven, studies have demonstrated that MHC-I-restricted N-glycosylated epitopes can elicit highly efficient CD8 T cell responses [23]. Furthermore, a fundamental previous study has provided direct evidence that the proteasome is fully capable of degrading glycoproteins without prior removal of their glycans [32]. While the addition of *N*-linked glycans always occurs within the ER, potential removal of the glycans takes place in the cytosol through the action of the peptide *N*-glycanase (PNGase). The same study also demonstrated that the presence of a glycan near an MHC-I-restricted epitope modulates its presentation [32].

Others and we have previously addressed from a structural point of view how specific viral escape mutations and/or PTM in MHC-I-restricted epitopes alter significantly T and NK-cell recognition [6,9,26,33–35]. In particular, the effects of glycosylation and nitrotyrosination on the formation of neo-epitopes that select for PTM-specific T cells were described [6,26]. Often, the same PTM in the same epitope reduced significantly binding capacity when restricted to one MHC allele, while forming a neo-epitope when binding to another MHC allele, allowing for both immune escape and selection of a novel CTL repertoire [6,9,26,34,35]. Furthermore, clear possibilities for TCR cross-reactivity were also suggested by at least one study [9].

The LCMV (Lymphocytic Choriomeningitis Virus)-derived epitopes GP92 (CSANNSHHYI) and GP392 (WLVTNGSYL) were initially identified and characterized by using peptide libraries based on the H-2D^b^ binding motif within LCMV protein sequences [36]. The sequences corresponding to these two epitopes are naturally glycosylated in the full length protein version [23,24,37]. More recently, the crystal structure of the prefusion surface glycoprotein, from which both these peptides are derived, demonstrated clearly that the asparagine residues at position 4 and 5 of GP92 and GP392, respectively, are both glycosylated [38] (S1 Fig). Thus, both epitopes carry the N-linked glycosylation pattern, with well-established functional effects [23,24]. However, the introduced glycosylation modifications have opposite effects on GP92 and GP392 epitopes. Indeed, the sub-dominant peptide GP92 and its modified forms GlcNAc-GP92 and D-GP92 are all immunogenic [23]. Vaccination with any of these three variants triggers efficient T cell activation in mice models but only CTLs generated with either wild-type GP92 or D-GP92 can kill LCMV-infected cells [23,24]. However, T cells raised against the GlcNAc-GP92 epitope cannot lyse LCMV-infected cells, indicating that this epitope is not naturally presented or that the amounts presented on the cell surface are very limited [23]. In contrast to GP92, GP392 is immunogenic only in its genetically-encoded form when used for vaccination in mice; T cells could not be raised towards neither GlcNAc-GP392 nor the de-glycosylated variant D-GP392 [24]. Interestingly, the binding affinity of the two modified versions of GP392 were significantly reduced compared to the unmodified GP392 [24]. Most importantly, neither unmodified GP392 nor the PTM GP392 epitope variants were detected on the cell surface of LCMV-infected cells, reducing the potential for using any of these epitopes for vaccination attempts [24].

The main aims of the present study were to determine the crystal structures of H-2D^b^ in complex with the LCMV-derived epitopes GP92 and GP392 and possibly unveil the structural mechanisms underlying the differential immunogenicity of these two epitopes and their respective glycosylated isoforms. To our knowledge, our results represent the first structural analysis of the differences introduced by peptide glycosylation on the immunogenicity of MHC-I-restricted LCMV-associated epitopes. Additionally, the two crystal structures solved within this study provide further insights on the potential effects of PTMs on viral immune escape from cytotoxic T Lymphocytes (CTL) responses as well as how PTM can generate immunogenic neo-epitopes.

## Materials and methods

### Production and crystallization of H-2D^b^ in complex with GP92 and GP392

Peptides GP92 (CSANNSHHYI) and GP392 (WLVTNGSYL) were produced by microwave-assisted solid phase synthesis based on Fmoc chemistry [39] on a CEM Liberty peptide synthesizer. Peptides were purified by RP-HPLC on a Jasco BS-997-01 instrument equipped with a DENALI C-18 column from GRACE VYDAC (10μm, 250 x 22mm). The refolding and purification of H-2D^b^ in complex with GP92 or GP392, and mouse β_2_-microglobulin (β_2_m) were conducted as described earlier [6,34,40–43]. Ion-exchange chromatography was used as an additional purification step, using a Mono Q column (GE Healthcare), for both complexes. The best crystals for the H-2D^b^/GP92 and H-2D^b^/GP392 complexes were obtained by hanging drop vapor diffusion in 100 mM ammonium sulfate, 100 mM Tris-HCl (pH 7.5), 25% PEG 6000 and 1.8 M ammonium sulfate, 100 mM Tris-HCl (pH 8.0), respectively. Typically, 2 μl of H-2D^b^/GP92 (2 mg/ml) and 4 μl of H-2D^b^/GP392 (5 mg/ml), both in 100 mM Tris-HCl, pH 7.5, were mixed at a 2:1 ratio with the crystallization reservoir solution at 4°C.

### Data collection and processing

Crystals were soaked in reservoir solutions complemented with 15% glycerol before flash-freezing in liquid nitrogen. Data sets for the H-2D^b^/GP92 and H-2D^b^/GP392 complexes were collected at a wavelength of 0.933 Å under cryogenic conditions (temperature 100 K) at the beamline ID14-1 at the European Synchrotron Radiation Facility (ESRF Grenoble, France) to 2.4Å and 2.5Å resolution, respectively. The data were processed with MOSFLM [44] and SCALA [45] from the CCP4 suite [46]. While the space group of the H-2D^b^/ GP92 crystal was determined to be P2_1_ with four molecules in the asymmetric unit, the crystal of H-2D^b^/GP392 belongs to the space group C2 with one molecule in the asymmetric unit. Data collection statistics are provided in Table 1.

**Table 1:** Data collection and refinement statistics.

	*H-2D^b^/CSANNSHHYI*	*H-2D^b^/WLVTNGSYL*
***Data Collection***		
*Space group*	P2_1_	C2
*Unit-cell parameters (Å, °)*	a = 85.5, b = 176.3, c = 85.6	a = 90.3, b = 108.8, c = 58.0
	α = γ = 90, β = 119.8	α = γ = 90, β = 121.9
*Resolution*	19.61–2.4 (2.43–2.4)	19.76–2.5 (2.66–2.5)
*^1^R_merge_*	7.0 (69.0)	10.0 (40.3)
*I/σ(I)*	12.7 (1.8)	12.2 (3.4)
*Completeness (%)*	94.2 (91.0)	99.5 (99.2)
*Multiplicity*	3.6 (3.6)	3.6 (3.6)
		
***Refinement***		
*No. of reflection*	84550 (2598)	16374 (2590)
*^2^R_work_*	0.1994 (0.3476)	0.1801 (0.2523)
*^3^R_free_*	0.2589 (0.3997)	0.2291 (0.2857)
		
***R.m.s.d***		
*Bond lengths (Å)*	0.010	0.010
*Bond angles (°)*	1.241	1.202
		
*Ramachandran outliers*	0	0
*Ramachandran favored*	97.81%	0,97
*Mean B factor (Å^2^)*	67.87	58.0

Values in parentheses are for the highest resolution shell.

^1^ R_merge_ = ∑_hkl_ ∑i │Ii (hkl)—‹I (hkl)›│/ ∑_hkl_ ∑i Ii (hkl), where Ii(hkl) is the ith observation of reflection hkl and ‹I (hkl)› is the weighted average intensity for all observations i of reflection hkl.

^2^ R_work_ = Σ||Fo|—|Fc||/Σ|Fo|, where |Fo| and |Fc| are the observed and calculated structure factor amplitudes of a particular reflection and the summation is over 95% of the reflections in the specified resolution range. The remaining 5% of the reflections were randomly selected (test set) before the structure refinement and not included in the structure refinement.

^3^ R_free_ was calculated over these reflections using the same equation as for R_cryst_.

### Structure determination and refinement

The crystal structures of the H-2D^b^/GP92 and H-2D^b^/GP392 complexes were solved by molecular replacement using the program PHASER [47] and the crystal structure of H-2D^b^/gp33 [34] (PDB code 1N5A), with the peptide omitted, as a search model. Clear and continuous electron densities were observed in the peptide binding clefts in both structures, allowing for unambiguous modelling of the epitopes CSANNSHHYI and WLVTNGSYL. Five percent of the reflections were set aside in both cases for use as a test set for cross-validation. Refinements were carried out using Phenix [48] and manual rebuilding using Coot [49]. The final refined structures were deposited at the PDB under the accession codes 5JWD and 5JWE for GP392 and GP92, respectively. The final refinement parameters are presented in Table 1. All figures were generated using Pymol [50].

## Results

### The 3D structures of H-2D^b^/GP92 and H-2D^b^/GP392 are prototypic for MHC-I/peptide complexes

**Fig 1:**
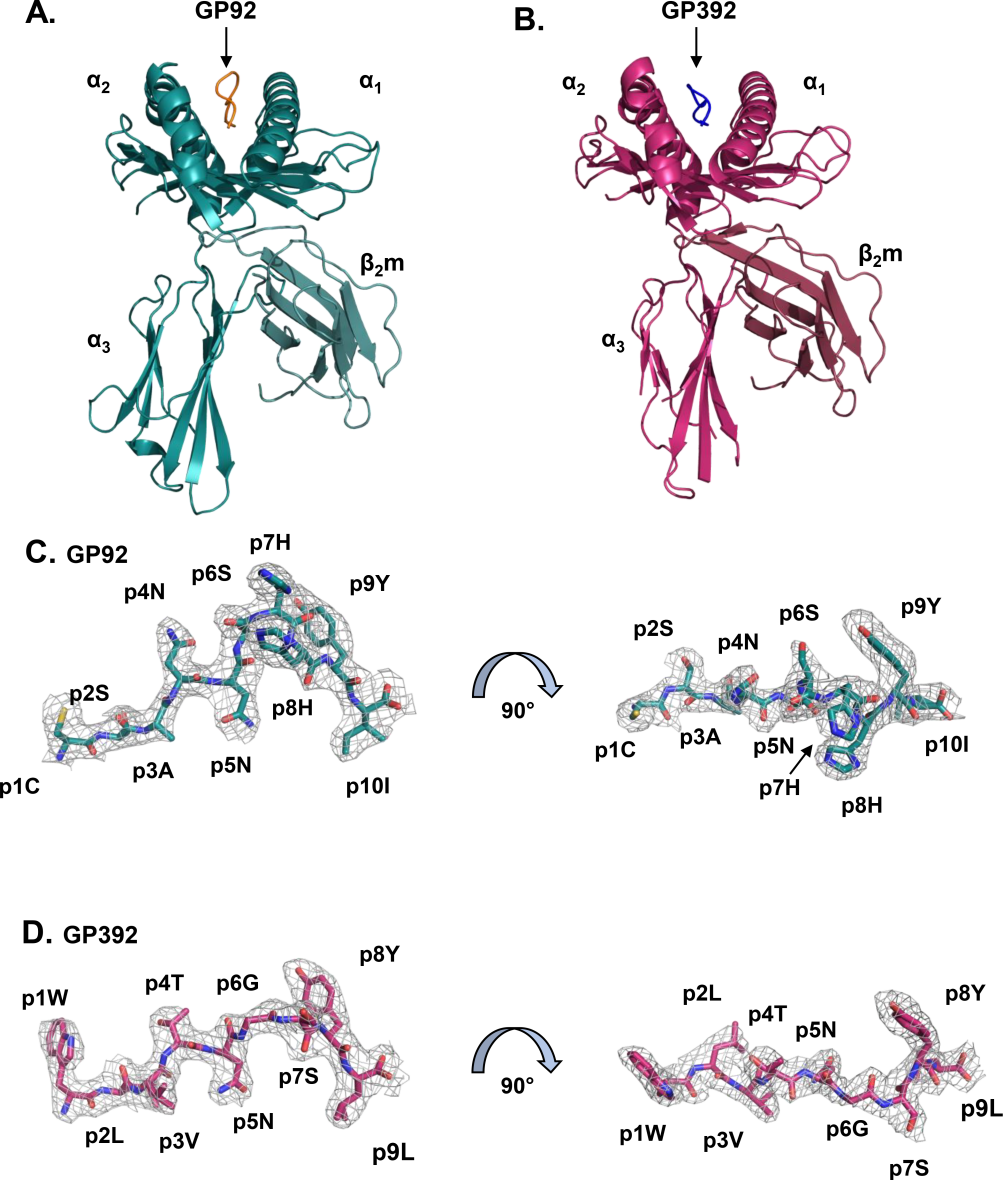
H-2D^b^/GP92 and H-2D^b^/GP392 have highly similar overall fold. Cartoon representations of the extracellular domains of the H-2D^b^/GP92 complex (**A**) with the peptide in orange and the H-2D^b^/GP392 complex (**B**) with peptide in blue demonstrate classical MHC-I overall structures, with the peptides bound between the helices of the α_1_ and α_2_ domains. (**C and D**) 2FoFc electron density map contoured at 1σ level defines unambiguously the conformation of the peptides. Peptides are shown in stick representation with N-terminus to the left. (**C**) The decameric GP92 takes a bulged conformation when binding to the H-2D^b^ cleft. Residues p7H and p8H project towards the solvent and, together with p4N and p9Y, protrude towards the TCR. The side chains of residues p5N and p10I anchor the peptide to H-2D^b^. (**D**) The epitope GP392 takes an elongated conformation with residues p5N and p9L anchoring to the peptide binding cleft and residues p4T and p8Y directed towards the TCR.

The crystal structures of H-2D^b^ in complex with the LCMV-derived peptides GP92 (CSANNSHHYI) and GP392 (WLVTNGSYL) were refined to 2.4 and 2.5 Å resolution, respectively (Table 1). R_free_/R_cryst_ for the final models of GP92 and GP392 were 25.9/19.9% and 23.5/21.6%, respectively, which is typical for structures at these resolutions. The overall structures of the complexes are very similar to each other and display the classical configuration for MHC-I/peptide complexes (Fig 1A and 1B) with both epitopes bound within the peptide binding cleft [51]. The peptide-binding domains of these two MHC-I/peptide complexes are highly similar to each other with an overall root mean square deviation (rmsd) value of 0.78Å for 170 Cα atoms (residues 3–173) in the α_1_ and α_2_ domains of the heavy chain. Moreover, their three-dimensional structures are very similar to previously determined crystal structures of H-2D^b^ in complex with *e*.*g*. the immunodominant LCMV-derived peptide gp33 [35], with an rmsd value of 0.66Å for H-2D^b^/GP92 and 0.87Å for H-2D^b^/GP392, respectively. The stereochemistry of both crystal structures is as expected for models at these resolutions (Table 1). The bound peptides and all residues forming the binding cleft of H-2D^b^ are clearly defined in unambiguous 2Fo-Fc electron density maps (Fig 1C and 1D).

### The LCMV-derived epitopes GP92 and GP392 bind conventionally to H-2D^b^

**Fig 2:**
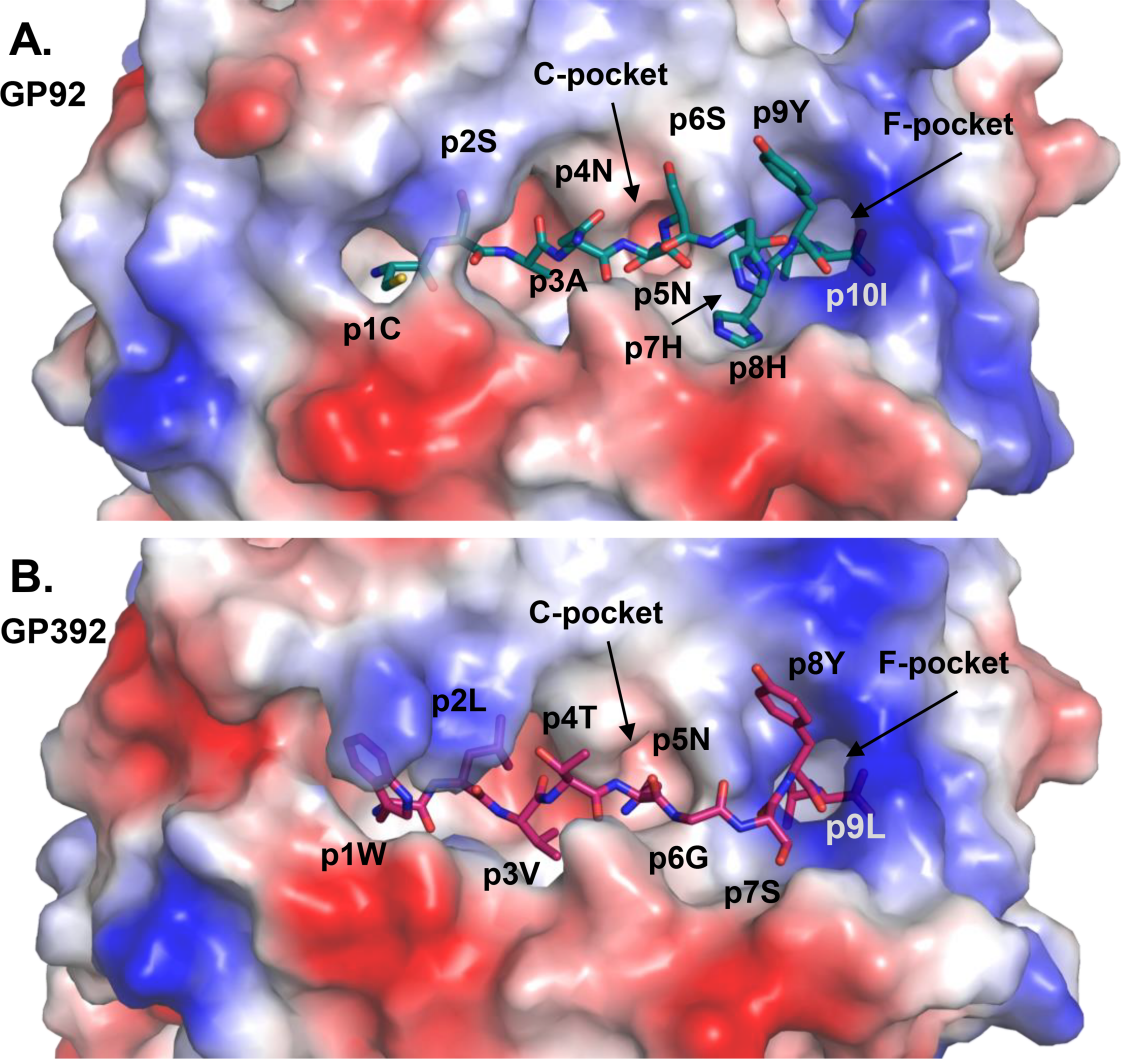
The peptides GP92 and GP392 bound to the cleft of the H-2D^b^ molecule. Peptides are presented as sticks and the H-2D^b^ molecule is shown as a surface colored according to the electrostatic potential with red and blue corresponding to negative and positive charges, respectively. The C- and F-pockets, docking sites for the anchor residue p5N and the C-terminal residue, respectively, are indicated.

**Fig 3:**
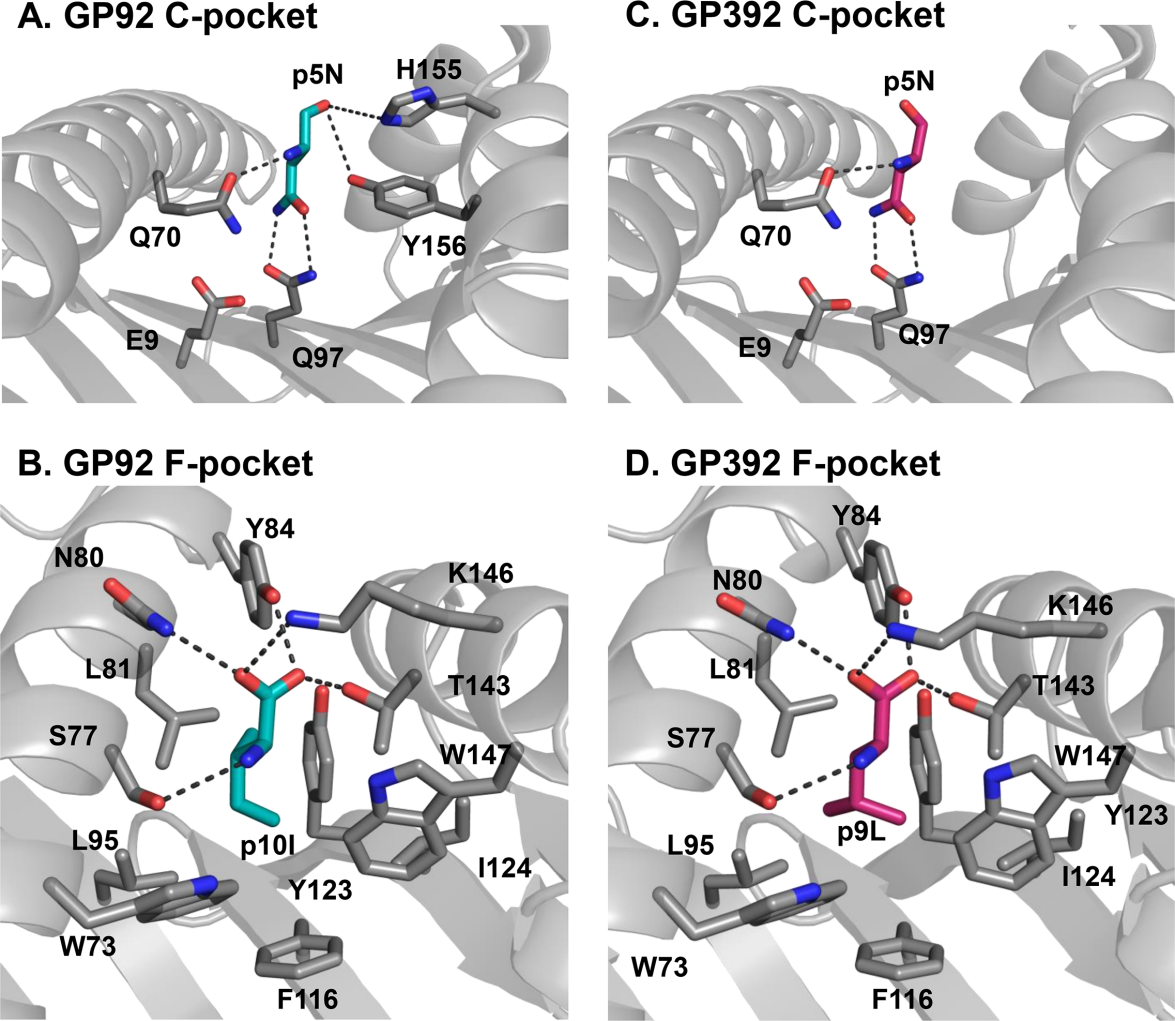
The two main anchor residues are using hydrogen bonds and hydrophobic interactions for binding to H-2D^b^. H-2D^b^ residues shown in grey interact with anchor residues of GP92 (cyan) (**A-B**) and GP392 (magenta) (**C-D**). The conformation and the interaction of residue p5N in GP92 (**A**) and GP392 (**C**) are almost identical. Residue E9 is responsible for the slightly negative charge of the pocket C, impairing docking of *e*.*g*. aspartate residues in the C-pocket. The heavy chain oxygen of peptide residue p5N in GP92 is directed towards the H-2D^b^ residues H155 and Y156, forming hydrogens bonds. In contrast, the oxygen residue in GP392 does not form any interaction with H155 nor Y156. The details of the interactions of the GP92 and GP392 C-terminal residues p10I and p9L within the F-pocket are presented in (**B**) and (**D**), respectively. Though isoleucine and leucine residues are rather similar in size and hydrophobicity, the shape of the F pocket fits better for isoleucine residue p10I (**B**) rather than leucine (**D**).

The peptide-binding groove of H-2D^b^ is closed at both ends restricting most bound peptides to 8–10 residues. In both complexes, the fifth peptide residue (asparagine p5N) and the C-terminal residue act as anchor positions, binding to the C- and F-pockets in H-2D^b^, respectively [52]. Indeed, the decamer GP92 employs p5N and p10I as main anchor residues for binding to H-2D^b^. A small section of the C-terminal part of GP92, composed of residues p6S-p9Y, bulges out from the groove in order to accommodate the anchor residue p10I within the F-pocket (Figs 1C and 2A) [53]. The other main anchor residue p5N forms two hydrogen bonds with the H-2D^b^ residue Q97, and an additional hydrogen bond with the H-2D^b^ residue Q70 (Fig 3A). The side chain of residue p10I fits well within the hydrophobic F-pocket of H-2D^b^ composed by residues W73, L81, L95, F116, I124 and W147 (Fig 3B). Its main chain atoms also form an intricate network of hydrogen bonds with the H-2D^b^ residues S77, N80, Y84, T143 and K146 (Fig 3B). All other peptide residues, except the bulging histidine residue p7H and non-anchoring residue p3A, form hydrogen bonds between their backbone atoms and the side chains of H-2D^b^ residues (S2 Fig).

The nonameric GP392 binds to the peptide binding cleft in a classical elongated conformation (Figs 1D and 2B). Similarly to GP92, GP392 utilizes residues p5N and p9L as main anchor residues (Fig 3C and 3D). Residues p5N and p9L use approximately the same hydrogen bond pattern as GP92, besides two hydrogen bonds that are missing due to a shift in the main chain oxygen of residue p4N causing the distance to H155 and Y156 to increase. It should be noted that the leucine residue p9L is considered as a weaker anchor in the F-pocket, compared to strong anchors such as isoleucine or methionine, with a following reduction in hydrophobic interactions [53].

### The glycan-binding asparagine residue is exposed in GP92 and buried in GP392

**Fig 4:**
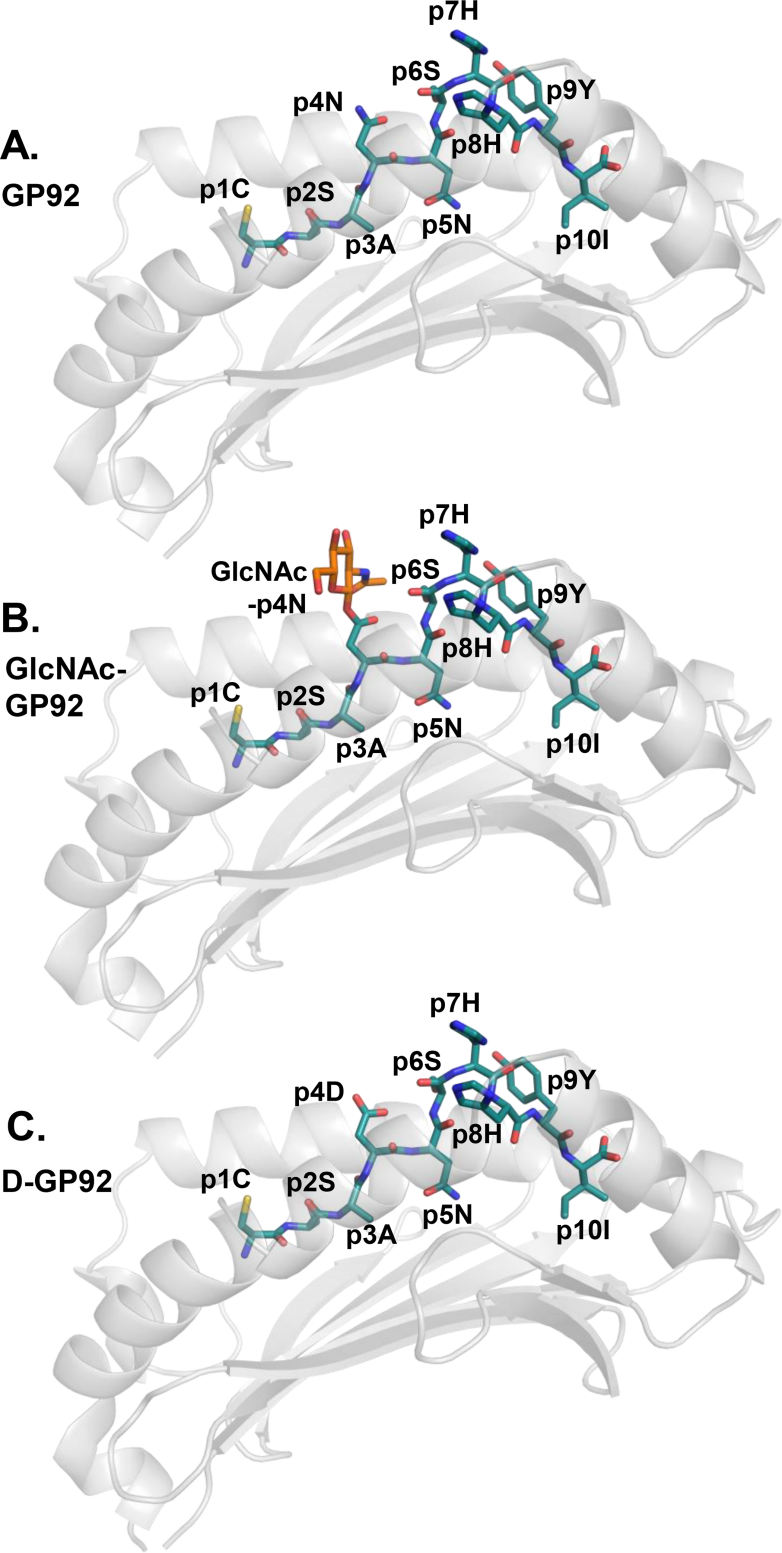
Molecular models of glycosylated and de-glycosylated GP92 indicate the possible formation of neo-antigens following PTM. (**A**) The crystal structure of the H-2D^b^/GP92 complex indicates that residues p4N and p7H are likely important for interactions with TCRs. (**B**) Potential glycosylation of p4N in GlcNAc-GP92 would result in the creation of a neo-antigen. (**C**) Deamidation of p4N in D-GP92 would also result in the formation of a neo-epitope potentially recognized by a different TCR population compared to the wild-type epitope.

In GP92, residue p4N that can be glycosylated protrudes towards the solvent and is fully accessible to T cell receptors (TCRs) (Fig 4A). Thus, in line with the observations of Hudrisier *et al* [24], modifications targeting this residue should not significantly affect the binding capacity of the altered peptide(s) to H-2D^b^. As previously mentioned, vaccination with wild-type or any of the two PTM GP92 variants generated T cells selectively cytotoxic to LCMV-infected cells [23]. Based on the crystal structure of H-2D^b^/GP92 solved in this study, we created molecular models of the different PTM versions of GP92 known to bind to H-2D^b^ (Fig 4B and 4C), indicating that both the glycan moiety at p4 and the modified residue p4D protrude from the cleft, and explaining the functionally observed efficient T cell triggering for all three GP92 peptide variants [23]. Indeed, as T cells activated by GlcNAc-GP92 or D-GP92 cannot efficiently cross-react with the wild-type epitope [23], both the wild-type epitope and each PTM version are most probably recognized by different TCR populations.

**Fig 5:**
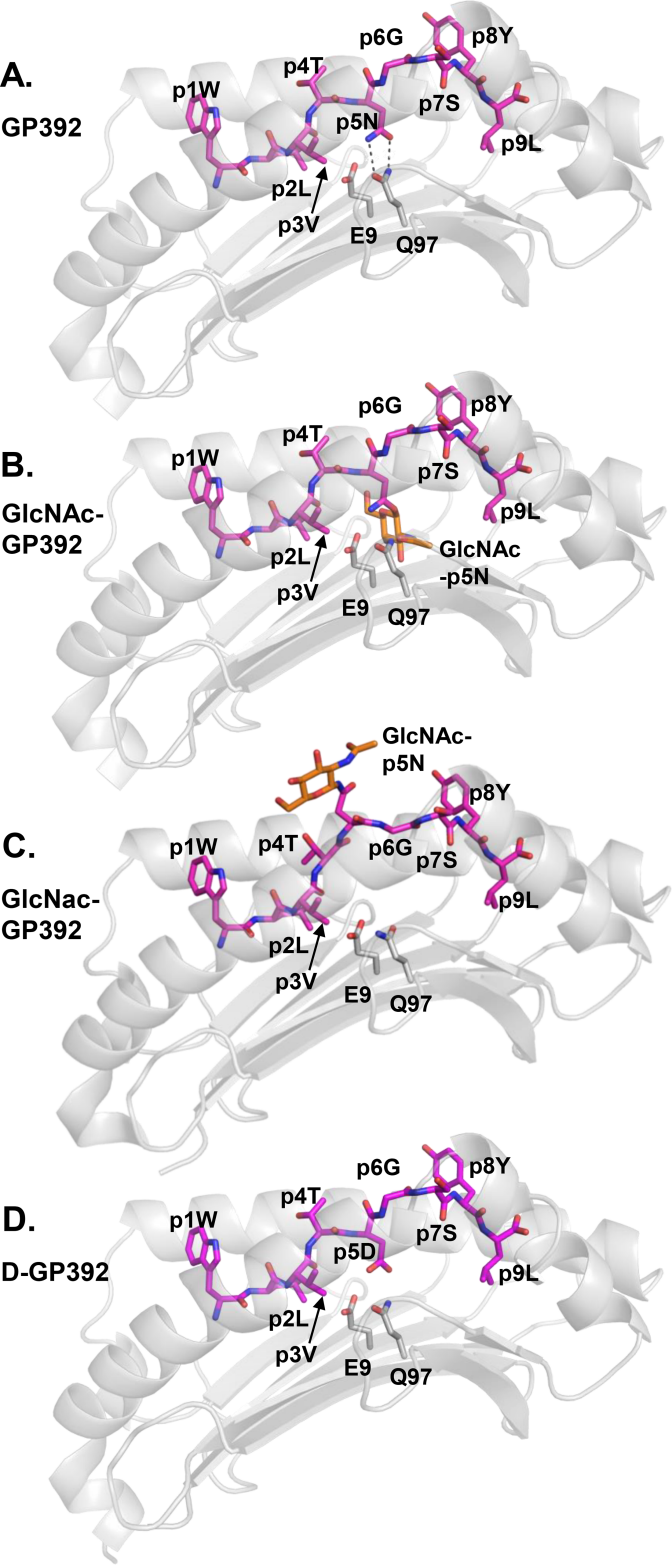
Molecular models of glycosylated and de-glycosylated GP392 explain reduced binding to H-2D^b^ as well as possibilities for the formation of neo-epitopes. (**A**) The main anchor residue p5N in the unmodified GP392 forms hydrogen bonds with residue Q97 localized in the bottom of the peptide binding cleft of H-2D^b^. (**B**) Molecular modeling indicates that the introduction of the smallest possible sugar moiety GlcNAc induces sterical clashes that impairs binding. (**C**) Molecular modeling also indicates that glycosylation of p5N could result in profound conformational modifications within the middle part of the epitope, that could allow presentation of the glycan moiety towards the TCR. (**D**) The negatively charged p5D residue in the de-glycosylated D-GP92 is directly unfavorable for binding to the C-pocket, due to incompatibilities with the H-2D^b^ residues E9 and Q97.

In contrast to GP92, the crystal structure of H-2D^b^/GP392 indicates that the asparagine residue p5N that can be glycosylated is essential for peptide binding to H-2D^b^ (Figs 1D, 3C and 5A). Addition of the sugar not only disrupts the fork-like hydrogen bonds formed between p5N and the H-2D^b^ residue Q97, but also transforms the glycosylated p5N position into a much larger residue unsuitable for binding into pocket C (Figs 3C and 5B). However, previous studies have also demonstrated that the glycosylated version of GP392 still can bind to H-2D^b^, although with much weaker affinity compared to the wild-type GP392 [24]. Furthermore, other crystal structure studies of glycosylated peptides in complex with H-2D^b^ have demonstrated that a glycan at peptide position 5 such as in the epitope K2G protrudes out of the cleft instead of pointing towards the H-2D^b^ residue Gln97 [9]. Consequently, the K2G peptide takes a profoundly different conformation in its middle part while both the N- and C-termini of the peptide take very similar conformations compared to the wild-type peptide. Here instead, the tyrosine residue at peptide position 6 in K2G acts as a novel anchor position, resulting in efficient binding to H-2D^b^ [9]. Therefore, we also created a molecular model of H-2D^b^ with GlcNAc-GP392 that indicates that the glycosylated residue at position 5 can also point out of the cleft (Fig 5C). However, the glycine residue at position 6 cannot act as an anchoring position substitute as in the complex of H-2D^b^ with K2G, providing a possible explanation to its much weaker affinity to H-2D^b^, compared to GP392.

It is well-established that any variation in anchor residues may alter considerably the capacity of modified peptides to bind to MHC-I [54]. The molecular model of the deglycosylated peptide variant D-GP392 demonstrates this clearly (Figs 3C and 5D), since the single atom difference between aspartate and asparagine, abrogates interactions with Q97 and results in a significant reduction in peptide affinity to H-2D^b^ [24]. Analysis of the crystal structure also reveals the presence on the bottom of the peptide-binding cleft of the negatively charged residue D9 (Fig 3C), which renders pocket C (also formed by residues Q70 and Q97) more acidic. Thus, pocket C is a perfect docking site for polar uncharged asparagine residues whereas modification to a negatively charged aspartate is unfavorable. Overall, our results demonstrate that glycosylation may both reduce significantly binding affinity and thus presentation capacity and/or alter the conformation of the presented epitopes, possibly allowing for immune escape and/or forming a neo-epitope that may select for a different set of CD8 T cells.

## Discussion

Post-translational modifications affect the characteristics of diseases including *e*.*g*. cancers [12–14], autoimmune disorders [15,16] and viral infections [17,18]. Even though these studies have demonstrated their clear impact on these diseases, the contrasting effects of PTMs on immunogenicity and binding affinity require further investigations in order to progress epitope vaccination design. Indeed, glycosylation, one of the most common PTMs, has significant impact on MHC-I epitope repertoire and immunogenicity [13]. We based the present structural analysis on the LCMV-derived H-2D^b^-restricted epitopes GP92 and GP392 which both comprise the classical motif for glycosylation. However due to the different location of these sites on GP92 and GP392, glycosylation results in diametrically different effects on peptide binding and immunogenicity in these two different peptide models [24].

GP92 and GP392 share p5N as a primary anchoring residue, important for binding to H-2D^b^ due to its polarity and the formation of a hydrogen bond network with H-2D^b^ residues inside the C pocket (Fig 3A and 3C). The majority of the deposited crystal structures and peptides eluted from H-2D^b+^ cells comprise an asparagine at position 5 in the peptides as preferred anchor residue for C pocket [53] with exceptions such as the recently identified cancer-associated TEIPP neo-epitope Trh4, that is presented exclusively on antigen processing deficient cells [55–57], and a mutated version of the influenza A epitope NP_366_ [58]. Accordingly, any PTM introduced at position 5 in GP392 will have significant consequences for its binding ability to H-2D^b^. Thus if pointing down towards the peptide-binding cleft, this sugar moiety should prevent/reduce peptide binding (Fig 5B), explaining the significant reduction in binding and immunogenicity in vaccination trials [24]. On the other hand, as previously demonstrated [9], PTM at peptide position 5 can result in profound conformational modifications within the middle part of H-2D^b^-restricted epitopes, resulting in the presentation of the glycan towards TCR and away from the peptide-binding cleft. Indeed, molecular modeling of H-2D^b^ in complex with GlcNAc-GP392 indicates also the possibility for the glycan moiety to be presented at the TCR interface through a significant conformational modification in the central part of the epitope (Fig 5C). In contrast to previous studies, the glycine residue at position 6 of GP392 cannot compensate for the lost interactions with the H-2D^b^ residue Gln97. In conclusion, this alternative conformation could result in the creation of a neo-antigen. Furthermore, it could also explain the reduced binding ability of the glycosylated peptide. The altered peptide variant D-GP392 (Fig 5D) differs from GP392 in only one atom, the oxygen OD1 instead of the nitrogen ND1. Nonetheless, D-GP392 binds to H-2D^b^ with a significantly reduced affinity of 2–3 order magnitude compared to the wild-type epitope [24].

Though the unmodified GP392 is a promising candidate for binding and presentation [53], it has hitherto not been detected on the surface of LCMV-infected cells [24]. It is now well established that this epitope is glycosylated in the native protein [24,38]. It should be noted that the consensus sequence Asn-X-Ser/Thr is not the only parameter for an effective N-glycosylation; it has indeed also been shown that the N-linked glycan occupancy is dependent on the conformation of the glycosylated part of the protein [59]. Several possibilities may explain the fact that presence of glycosylated GP392 on the cell surface has hitherto not been demonstrated. One possibility is that GlcNAc-GP392 is completely de-glycosylated by PNGase following protein degradation by the proteasome, allowing only the presentation of D-GP392. Another possibility is that due to the significantly reduced binding affinity of GlcNAc-GP392 to H-2D^b^, with the glycan moiety pointing either in or out of the peptide-binding cleft, the amounts of GlcNAc-GP392 presented on the cell surface are too low for efficient detection. It is essential to note that *in vivo* peptide stimulation using N-glycosylated and de-N-glycosylated versions of GP392 failed to generate CTL responses in C57/Bl6 mice [24].

In contrast to GP392, the GP92 epitope is not modified at position 5, but instead at position 4 (Fig 4A), causing peptide binding to be largely unaffected by glycosylation or de-glycosylation (Fig 4B and 4C). Indeed all forms of GP92 epitopes efficiently bind to H-2D^b^ [24]. Instead, glycosylation and any following modification would result in the formation T-cell neo-epitopes [23]. Formation of glycosylation/de-glycosylation derived neo-epitopes has been reported in a large ensemble of diseases including ovarian carcinoma, melanoma, leukemia as well as autoimmune diseases such as rheumatoid arthritis [12,13,15,60].

The epitope GlcNAc-GP92 is immunogenic with high affinity to H-2D^b^, but has not been eluted from LCMV-infected cells, and CTLs stimulated with GlcNAc-GP92 epitope did not kill LCMV-infected cells. [23]. In contrast, both wild-type GP92 and the de-glycosylated PTM version D-GP92 are naturally presented on the cell surface. So why is GlcNAc-GP92 not present? At the present time, we can only delve into this question through speculation. Glycosylation is known to affect the efficiency of proteases [20], which could be one reason to why only the wild-type and the de-glycosylated peptide versions are detected. Assuming that the glycosylated version is the functional state of this protein, the H-2D^b^-restricted peptide GP92 could be a result of a misfolded and degraded protein, insusceptible to glycosylation at this position, according to the Defect Ribosomal Product hypothesis [61].

Additional studies on the functional, structural and immunological relevance of the post-translationally modified peptidome are necessary in order to expand our knowledge on the pathways leading to the presentation of such peptides. Novel techniques using *e*.*g*. advanced mass-spectrometry with high sensitivity [16,60,62,63] would allow the identification of disease-associated PTM neo-epitopes, and allow us to readdress previously published work in order to assess whether glycosylated versions of GP92 are actually presented or not on the surface of infected cells. Here we report the structural bases underlying how glycosylation of GP92 can clearly result in the formation of immunogenic neo-epitopes that may select for different T cell populations. In contrast, glycosylation of GP392 inhibits the presentation of variants of this epitope, possibly resulting in immune evasion. In conclusion, our results provide structural insights for how PTM neo-epitopes can be immunogenic while others are not. The identification and exact understanding of the structural localization of such modifications, especially when it comes to interaction with MHC-I molecules, is essential for the design of efficient peptide vaccines against viral infections and cancer.

## Supporting information

S1 Fig Crystal structure of the prefusion surface glycoprotein of LCMV expressed in insect cells reveals that GP is heavily glycosylated. Eight asparagine residues, N85, N95, N114, N124, N171, N232, N371 and N396 glycosylated. Importantly bothpeptide GP92 and GP392, colored in green and blue, respectively, are glycosylated.
(TIF)

S2 Fig Extensive network of hydrogen bonds between peptide and H-2D^b^. (TIF)

## Abbreviations

ER: endoplasmic reticulum
LCMV: lymphocytic choriomeningitis virus
MHC-I: MHC class I
pMHC: MHC class I/peptide complex
CTL: cytotoxic T lymphocyte
GlcNAc: N-acetyl-glucosamine
