# Supplementary figures and images for: Crystal structures of H-2Db in complex with the LCMV-derived peptides GP92 and GP392 explain pleiotropic effects of glycosylation on antigen presentation and immunogenicity
Source: PLoS One. 2017 Dec 18;12(12):e0189584. doi: 10.1371/journal.pone.0189584 (PMC5734757; doi:10.1371/journal.pone.0189584)

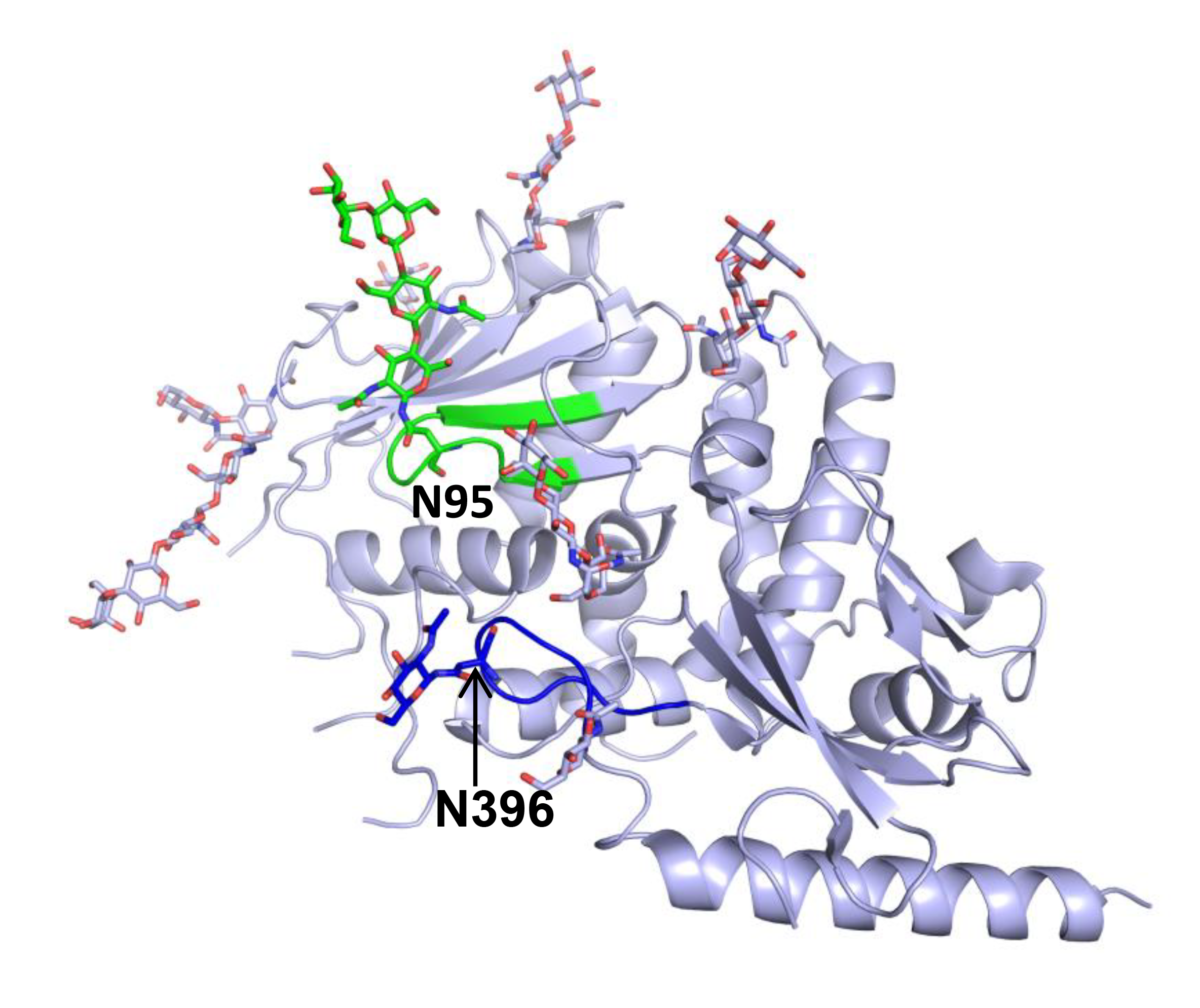

Supplement: S1 Fig — Eight asparagine residues, N85, N95, N114, N124, N171, N232, N371 and N396 glycosylated. Importantly bothpeptide GP92 and GP392, colored in green and blue, respectively, are glycosylated. (TIF) [file pone.0189584.s001.tif]

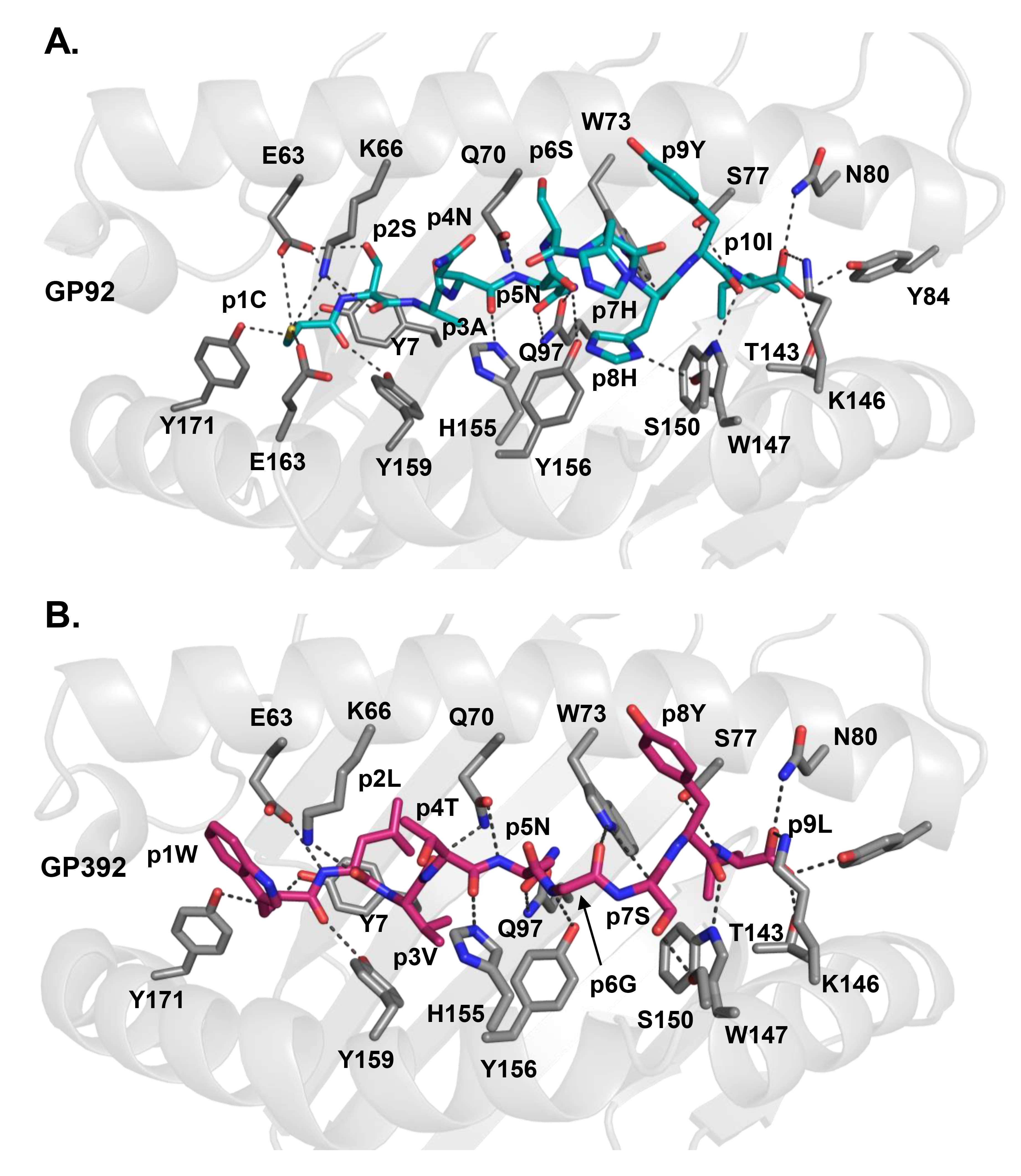

Supplement: S2 Fig — (TIF) [file pone.0189584.s002.tif]
